# Clinicoradiological Outcome of Concomitant Fractures of Proximal Femur and Femoral Shaft Treated With Second-Generation Cephalomedullary Nailing

**DOI:** 10.7759/cureus.15381

**Published:** 2021-06-01

**Authors:** Anurag Baghel, Kumar Keshav, Amit Kumar, Pulak Sharma

**Affiliations:** 1 Orthopaedics, Sanjay Gandhi Postgraduate Institute of Medical Sciences, Lucknow, IND

**Keywords:** ipsilateral, concomitant, hip, femoral shaft, femoral neck, intertrochanteric, fracture, cephalomedullary nail, proximal femoral nail, pfn

## Abstract

Background

The ideal modality of treatment of concomitant fractures of the hip (intertrochanteric/femoral neck) and the femoral shaft is still evolving. The aim of our retrospective study was to assess the clinicoradiological outcome of such fractures managed by closed second-generation cephalomedullary nailing.

Methodology

The study was conducted among skeletally mature patients presenting within one week of injury who underwent closed second-generation cephalomedullary nailing (proximal femoral nail). Those presenting beyond one week or those who had pathological fractures, incomplete follow-ups, or other modes of fixation were excluded. Functional results were evaluated according to Friedman and Wyman’s clinical assessment system. Time required for fracture healing and the presence of any complications were also noted.

Results

A total of 10 patients with the ipsilateral hip (five intertrochanteric and five femoral neck) and femoral shaft fractures were included in the study. Associated injuries found included fractures of the ipsilateral tibia/fibula at varying levels in three patients; hand and wrist injuries in two patients; and contralateral femoral shaft fracture, ipsilateral patella, bilateral crush injury of the foot, and head and chest injury with brachial plexus injury in one patient each. Four patients were diagnosed with intra-articular knee injuries (ligamentous and meniscal injuries) postoperatively. At the final follow-up, the functional outcome results were good in four, fair in one, and poor in five patients. All femoral neck fractures united at a mean of 15.2 weeks (range: 12.0-18.0 weeks) and intertrochanteric fractures at a mean of 14.0 weeks (range: 12.0-22.0 weeks). However, there was residual varus malunion in two intertrochanteric fractures. Eight femoral shaft fractures were infra-isthmic; of these, four resulted in nonunion (two of hypertrophic and two of atrophic type) and two were found to be in the delayed union, which eventually united by 24 weeks.

Conclusions

Second-generation cephalomedullary nail is an acceptable, cost-effective, and minimally invasive alternative for the management of concomitant ipsilateral fractures of the hip and supra-isthmic or isthmic femoral shaft fractures. For infra-isthmic fractures, retrograde femoral nail or distal femoral plate along with a separate implant for addressing the hip fracture (either cannulated cancellous screw or dynamic hip screw, preferably in a rendezvous/overlapping manner) are better options.

## Introduction

Concomitant fractures of the hip (intertrochanteric/femoral neck) and femur shaft are uncommon injuries [1-3]. These fractures are usually caused by high-energy trauma, such as road traffic accidents (RTAs) and falls from height. The incidence of RTAs is rising, and thus these fractures are being encountered more than ever before [1,4,5]. Currently, published studies place the rate of concomitant hip fractures among femoral shaft fractures at around 2.5%-6.0% [6,7]. Due to the advancement of diagnostic imaging modalities and improved understanding of the fracture patterns, the incidence quoted in recent literature is quite high [6-8]. Diagnosis of proximal fracture is often missed or delayed in approximately 30% of cases as these fractures are associated with polytrauma situations, and clinical signs of hip fractures are less obvious than those of other injuries [7,9,10].

These injuries have some interesting features. Femoral neck fractures are more commonly associated than intertrochanteric ones in a ratio of 3:1 [1,5,9]. The usual pattern of femoral neck fractures is basicervical, vertically oriented, and nondisplaced [11]. Femoral shaft fractures are often infra-isthmic, comminuted, found in the distal third segment, and associated with extensive soft tissue damage [1,2,4,10]. The injury mechanism in RTAs is often a dashboard injury with the hip flexed and abducted and the knee flexed (femoral head well-seated within the acetabulum). These injuries are more commonly seen among young adults [5,10-13]. The available literature indicates that the complications at the shaft level are higher than those around the hip. Additionally, in comparison to isolated hip fractures, the complications of proximal fractures in concurrent fractures are lower. As the first point of impact is always the knee, they are often associated with injuries in and around the knee [7,9,11,12,14-16].

A gold standard treatment is still not established in terms of correct implant choice, order of fracture management, and the timing of surgery. There are two schools of thought. One group of surgeons is in favor of inserting two implants for two fractures: a retrograde distal femoral nail (DFN) or a plate with cannulated cancellous screw (CCS)/dynamic hip screw (DHS). The other group prefers a single intramedullary implant. However, both procedures have their own pros and cons [6,8,13,14,16-20].

As this is a relatively rare injury pattern, only a few prospective studies with adequate sample sizes are described in the literature [7,13,21,22]. To enhance the literature on this rare pattern of injury, we conducted a retrospective study of hospital records at our tertiary care trauma center, the aim of which was to assess the clinicoradiological outcome of concomitant hip (intertrochanteric/femoral neck) fracture with femoral shaft fracture managed by closed second-generation cephalomedullary nailing. We also present a narrative review of the literature in regard to these fractures.

## Materials and methods

Our study, conducted at the Apex Trauma Centre, Sanjay Gandhi Postgraduate Institute of Medical Sciences, Lucknow, was a retrospective analysis of prospectively collected data of the cases operated on between July 2018 and March 2020 and followed for a minimum of one year. The inclusion criteria were skeletally mature patients (over 18 years old) with concomitant ipsilateral fractures of the hip (intertrochanteric/femoral neck) and femoral shaft with or without other injuries, who presented to us within one week of injury and underwent closed cephalomedullary nailing with a proximal femoral nail (PFN). Those presenting beyond one week of injury or those who had pathological fractures, incomplete follow-ups, or other modes of fixation were excluded.

Management protocol

All patients were managed primarily in the emergency area as per the standard Advanced Trauma Life Support protocol, followed by definitive management in the form of closed reduction and internal fixation by second-generation cephalomedullary nail (PFN) in the standard fashion [23]. However, there were a few specific steps that were required depending on the specific fracture pattern. First, we used a Schanz pin as the joystick for manipulation of the floating proximal trochanteric fragment and to reduce the proximal fracture (intertrochanteric/femoral neck). Provisional fixation was then done using two K wires, one passed anteriorly and one posteriorly in a manner that provided space for the passage of the nail [11,13,21,22]. Subsequent to the passage of the cephalomedullary nail (PFN), one or more screws (depending on the configuration of the nail) was passed in the femoral head.

Patients were started on quadriceps-strengthening exercises and encouraged to start knee mobilization the next day. Partial weight-bearing was started at six weeks, and then gradually full weight-bearing was begun after clinical/radiological evidence of a bridging callus. Patients were followed up at six weeks, three months, four and a half months, six months, nine months, and 12 months following surgical intervention until radiological union. Functional results were evaluated according to the clinical assessment system adopted by Friedman and Wyman [5]. The time required for fracture healing was recorded. Fractures were considered united if the anteroposterior and lateral radiographs showed three of four cortices with trabeculae bridging the fracture site and if the patient could bear weight without pain. We used the U.S. Food and Drugs Administration definition of nonunion as a fractured bone having not completely healed within nine months of injury and not shown progression towards healing over three consecutive months on serial radiographs [24].

## Results

A total of 10 patients who met the inclusion criteria were included in the study (Table 1). There were eight males and two females with a mean age of 36 years (range: 22-58 years). The average follow-up was 103 weeks (range: 66-123 weeks). All patients had suffered from high-energy trauma, with RTA as the mode of injury for nine patients and fall from height for one patient. Most patients had one or more associated injuries: fractures in the ipsilateral tibia/fibula at varying levels (three patients), in the contralateral femoral shaft (one patient), and in the patella (one patient); hand and wrist injuries (two patients); bilateral crush injury of the foot (one patient); and head and chest injury with brachial plexus injury (one patient). Injured patients were operated on within an average of six days (range: 2-18 days) after trauma. Four patients were diagnosed with intra-articular injuries (ligamentous and meniscal injuries) after fracture fixation during follow-up visits via clinical examination, which was confirmed with magnetic resonance imaging (MRI).

**Table 1:** Details of the patients included in the study. #: fracture; M: male; F: female; R: right side; L: left side; B/L: bilateral; AO: Arbeitsgemeinschaft für Osteosynthesefragen (German Working Group for Bone Fusion Issues); IT: intertrochanteric; ACL: anterior cruciate ligament; PCL: posterior cruciate ligament

Sl. no. of the patient	Age (Years)/gender	Side	Hip fracture		Femoral shaft fracture; location/ AO type	Associated injuries	Follow-up (weeks)	Number of days after trauma when surgery was performed	Union status/union time (weeks)		Complications			Friedman and Wyman’s functional outcome assessment
			IT/neck	AO type for IT fracture; Garden’s type/ anatomical level for femoral neck fracture					Hip	Shaft	Hip	Shaft	Specific/Other complications	
1.	22/M	R	Neck	Garden-2/basicervical	Middle third; 32-B2	# Both bone leg (R)	123	10	16	20	No	No	No	Good
2.	27/F	L	Neck	Garden-2/basicervical	Distal third; 32-A2	# Femoral shaft distal third (R); (32-A3)	121	7	18	Atrophic nonunion	No	Nonunion	Screw failure; iatrogenic subtrochanteric fracture (R); pain in both thighs	Poor
3.	32/M	R	Neck	Garden-2/basicervical	Distal third; 32-B2	Compound # proximal tibia (R); wrist dislocation (R); brachial plexus injury (R); head injury	121	18	18	24	No	Delayed union	Varus tibia; brachial plexus palsy still present	Poor
4.	58/M	R	IT	31-A1.2	Middle third; 32-B3	ACL injury (R); medial meniscal injury (R)	114	3	12	Hypertrophic nonunion	Varus malunion	Nonunion	Varus malunion of IT fracture; shortening of 2 cm; knee stiffness; distal screw failure	Poor
5.	27/M	R	Neck	Garden-2/basicervical	Distal third; 32-B3	# Second/third metacarpal (R) and proximal phalanx of index finger (R)	114	6	12	24	No	Delayed union	No	Good
6.	46/F	R	IT	31-A1.2	Distal third; 32-B3	Medial meniscus injury (R)	104	3	22	Atrophic nonunion	No	Nonunion	Shortening; medial meniscal injury; distal screw failure	Poor
7.	26/M	R	IT	31-A1.2	Distal third; 32-A3	Compound # proximal tibia (bicondylar) (R)	100	3	14	20	No	No	Mild knee stiffness and pain	Good
8.	29/M	R	Neck	Garden-3/transcervical/Pauwels Type 3	Distal third; 32-A2	# Patella (R) (undisplaced)	97	4	12	18	No	No	Mild knee pain (meniscal injury)	Good
9.	49/M	R	IT	31-A1.2	Distal third; 32-B2	PCL injury (R)	72	3	18	20	Proximal screw backout; varus malunion; heterotrophic ossification around hip	No	Knee instability; PCL reconstruction failure (fresh trauma)	Fair
10.	45/M	R	IT	31-A1.2	Distal third; 32-B2	# Distal tibia (R); crush injury foot (B/L)	66	4	18	Hypertrophic nonunion	No	Nonunion	Varus malunion of distal tibia fracture	Poor

Proximal (hip) fractures

**Figure 1:**
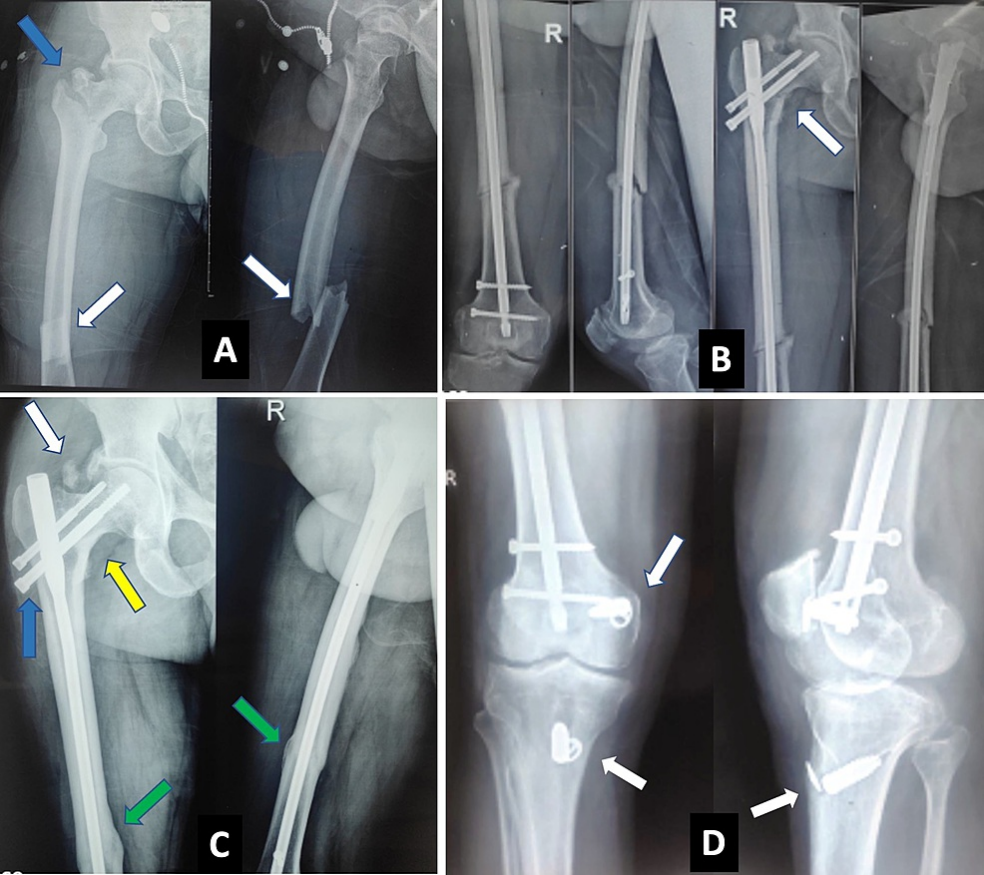
(A) Preoperative X-ray of a patient with intertrochanteric fracture (blue arrow) with femoral shaft fracture (white arrow) at the infra-isthmic level. (B) Immediate postoperative X-ray of the patient after cephalomedullary nailing showing fixation of intertrochanteric fracture in varus (white arrow). (C) Follow-up X-ray at 12 months showing union of the femoral shaft (green arrows). Heterotrophic ossification (white arrow), proximal screw backout (blue arrow), and varus malunion of the intertrochanteric fracture (yellow arrow) can also be seen. (D) The patient also had posterior cruciate ligament injury, for which he underwent reconstruction (white arrows). However, it failed later secondary to fresh trauma, leading to residual knee laxity.

A total of five patients had intertrochanteric fractures (Arbeitsgemeinschaft für Osteosynthesefragen [German Working Group for Bone Fusion Issues] [AO] Type 31-A1.2), and five had femoral neck fractures. Among the five femoral neck fractures, four were of Garden type 2/basicervical and one was of Garden type 3/transcervical/Pauwels type 3. All the femoral neck fractures united at a mean of 15.2 weeks (range: 12.0-18.0 weeks). Intertrochanteric fractures in our series united at a mean of 14 weeks (range: 12-22 weeks). However, two of these patients developed varus malunion, one of whom also had proximal screw backout and heterotrophic ossification around the hip (Figures 1A-1D). None of the cases in our series resulted in avascular necrosis (AVN) of the femoral head.

Femoral shaft fractures

Among the femoral shaft fractures, eight were in the distal third or junction of the middle and distal third (hence infra-isthmic) and two were in the middle third of varying types, as per AO classification. Among the patients whose fractures united, the average union time was 21 weeks (range: 18-24 weeks). Two cases were found to be in the delayed union, both of which eventually united in 24 weeks.

**Figure 2:**
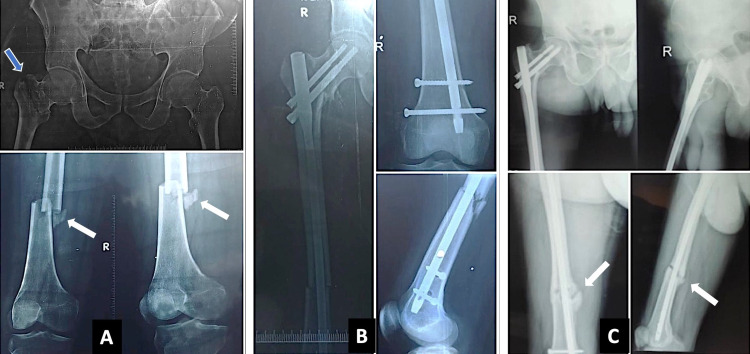
(A) Preoperative X-ray of one of the patients in our study showing intertrochanteric fracture (blue arrow) and infra-isthmic shaft fracture (white arrow). (B) Immediate postoperative X-ray of the patient after cephalomedullary nailing. (C) Follow-up X-ray at 12 months showing hypertrophic nonunion of the femoral shaft (white arrow).

Four femoral shaft fractures were not united at the end of the follow-up. Out of four cases of femoral shaft nonunion, two were of the hypertrophic type and two of the atrophic type. One such case is shown in Figures 2A-2C. All of these would require secondary procedures such as exchange nailing or nail removal with plating and autologous bone grafting. Due to the restrictions posed by the coronavirus disease 2019 pandemic, elective services have been stopped at our institute, and these patients are awaiting further surgery.

Functional outcomes

At the final follow-up, the functional outcome as per Friedman and Wyman’s clinical assessment system was good in four, fair in one, and poor in five patients. Of those who had a poor outcome, four were patients who developed femoral shaft nonunion. The fifth had a Gustilo type II compound fracture of the proximal tibia along with a head injury with brachial plexus injury. His brachial plexus injury has still not recovered, and his tibia has united in varus. One of the patients, who had a posterior cruciate ligament (PCL) injury, was operated on at another center; this patient eventually had a failure due to fresh trauma and is still having residual knee instability, giving him a fair outcome (Figures 1A-1D). All the patients had a functional range of movements at the hip.

Other complications

Screw failure was seen in four cases (proximal in one and distal in three). Knee pain with laxity (n = 2) or mild stiffness (n = 3) was not uncommon. Apart from the above patient with a PCL injury, the clinical presence of anterior cruciate ligament (ACL) injury was found in one patient and meniscal injuries in three, which were confirmed by MRI. However, the patients were able to continue with their normal routine activities and did not want further operative interventions as of the last follow-up. Some degree of terminal loss of motion was seen in a few other patients. The major findings of the study are summarized in Table 2.

**Table 2:** Summary of the major study findings. RTA: road traffic accident; AO: Arbeitsgemeinschaft für Osteosynthesefragen (German Working Group for Bone Fusion Issues); ACL: anterior cruciate ligament; PCL: posterior cruciate ligament

Variables	Outcome
Total number of patients	10
Age of patients	36 years (range: 22–58 years)
Male:female ratio	4:1
Mode of injury	RTA: 9
	Falls from height: 1
Side involved	Right: 9
	Left: 1
Associated injuries	Tibial plateau: 1
	Midshaft of tibia/fibula: 1
	Distal tibia: 1
	Contralateral femoral shaft: 1
	Patella: 1
	Hand and wrist: 2
	Bilateral crush injury foot: 1
	Head and chest injury with brachial plexus injury: 1
	ACL injury: 1
	PCL injury: 1
	Meniscal injuries: 3
Proximal (hip) fracture	Femoral neck: 5 (4 basicervical and 1 transcervical)
	Intertrochanteric: 5 (all were AO 31A1.2) (two-part simple pertrochanteric fracture)
Femoral shaft fracture location	Isthmic: 2
	Infra-isthmic: 8
Femoral shaft fracture AO type	32-A2 (simple oblique): 2
	32-A3 (simple transverse): 1
	32-B2 (intact wedge): 4
	32-B3 (fragmentary wedge): 3
Duration of follow-up	103 weeks (range: 66–123 weeks)
Functional outcome according to clinical assessment system adopted by Friedman and Wyman	Good: 4
	Fair: 1
	Poor: 5
Time required for union	Femoral neck fractures: 15.2 weeks (range: 12–18 weeks)
	Intertrochanteric fractures: 14 weeks (range: 12–22 weeks)
	Femoral shaft fractures: 21 weeks (range: 18–24 weeks)
Complications of proximal (hip) fracture	Varus malunion: 2 (among intertrochanteric fractures group)
	1 among them had proximal screw backout and heterotrophic ossification around the hip
Complications of femoral shaft fracture	Nonunion: 4 (hypertrophic: 2, atrophic: 2)
	Delayed union: 2
	Distal screw failure: 3

## Discussion

The salient findings of our study were the high incidence of femoral shaft nonunion. No complications were found with regards to femoral neck fractures. Few complications in the form of varus malunion and screw back-out were found with intertrochanteric fractures. We also observed an association with other injuries, especially those pertaining to the ipsilateral knee and leg. Functional outcomes varied greatly between patients depending upon the degree of the associated injuries and other complications.

Historically, fractures of the femoral neck, when found in association with femoral shaft fractures, are often delayed or missed in up to 30% of cases [11,14,25]. They are often (in 25%-60% of cases) nondisplaced at presentation [10,11]. In our series, we had one such case, which was detected intraoperatively, requiring the surgery plan to be changed from a conventional femoral interlocking nail to PFN. Tornetta et al. [10] proposed a protocol to identify femoral neck fractures, which consists of a dedicated anteroposterior internal rotation plain X-ray, a fine (2-mm) cut computed tomography (CT) scan through the femoral neck, an intraoperative fluoroscopic lateral radiograph prior to fixation, and finally, postoperative radiographs of the hip in the operating room before awakening the patient. Due to a high index of suspicion and improved imaging facilities, the incidence of missed injuries is decreasing.

Several case series have been published regarding the management of ipsilateral hip and femoral shaft fractures. Studies published in the last 15 years are summarized in Table 3 [6,7,11,13-19,21,22,25-27]. Some of the controversies in such fractures that remain to date are (i) single implant (cephalomedullary nail with or without additional screws) versus double implant (retrograde nail with DHS/CCS); (ii) order of fixation (whether hip fracture or shaft fracture should be fixed first); and (iii) acceptable reduction quality, especially in relation to the fractures of the femoral neck, and hence whether to use open reduction or closed reduction.

**Table 3:** Comparison of data from literature published in the past 15 years. #: fracture; M: male; F: female; R: right side; L: left side; B/L: bilateral; M: months; W: weeks; NA: not available; AO: Arbeitsgemeinschaft für Osteosynthesefragen (German Working Group for Bone Fusion Issues); IT: intertrochanteric; PFN: proximal femoral nail; CCS: cannulated cancellous screws; DHS: dynamic hip screw; PFNA II: proximal femoral nail antirotation II; ACL: anterior cruciate ligament; PCL: posterior cruciate ligament; AVN: avascular necrosis of the hip.

Serial number	Study	Year of publication	Number of patients	Mean duration of follow-up (Range)	Proximal # (femoral neck/intertrochanteric/subtrochanteric)	Femoral shaft # characteristics (location/type based on Winquist-Hansen classification/AO classification)	Implant used	Mean proximal # union time (Range)	Mean femoral shaft # union time (Range)	Clinical outcome (based on Friedman and Wyman assessment system, if mentioned)	Major complications
1.	Wei and Lin [25]	2021	22	12 M	Neck	Isthmic: 6; infra-isthmic: 16; Type A: 13 (A1: 1, A2: 2, A3: 10); Type B: 6 (B2); Type C: 3 (C1: 1, C2: 2)	Cephalomedullary nail with or without antirotation screw, DHS/CCS/bipolar hemiarthroplasty + retrograde femoral nail/plate	NA	NA	Only 8 (36.4%) had excellent or good results	Femoral neck malreduction: 1; femoral neck and shaft malunion: 1; plate breakage and femoral shaft nonunion: 1; femoral shaft hypertrophic nonunion: 9; femoral shaft atrophic nonunion: 1; femoral head AVN: 1
2.	Angelini et al. [15]	2021	9	4–36 M	Neck (2)/IT (5)/subtrochanteric (2)	NA	CCS/DHS with plate/long hip nail	3 (2–6) M	3 (2–6) M	7 united with good function	Death due to polytrauma: 1; femoral shaft nonunion: 2 (one of whom had thigh skin necrosis and the other one had plate breakage)
3.	Kang et al. [26]	2020	14	17.3 (9–30) M	Neck/IT	NA	Bridge link type combined fixation system	4.2 (3–6) M	5 (3–7) M	Good: 8; fair: 4; poor: 1 (FW)	Femoral neck nonunion: 1; femoral neck varus malunion: 1; femoral head AVN: 1; femoral shaft nonunion: 1; infection: 1
4.	Wu et al. [11]	2020	10	12 M	Neck	NA	PFNA II	NA	NA	NA	None
5.	Seong et al. [21]	2019	31	20.1 (12–48) M	IT (13)/subtrochanteric (18)	Isthmic: 18; infra-isthmic: 13	PFNA II/long PFN	16.2 (11–25) W	28.2 (12–52) W	Mean walking ability: 8.4 (7–9); Harris hip score: 90.7 (73–100)	Femoral shaft nonunion: 2
6.	Dahuja et al. [19]	2018	25	14.4 (6–24) M	Neck (18)/IT (7)	Proximal: 6; mid: 15; distal: 4	Long PFN	4.3 (3–6) M	5.4 (4–7) M	Good: 18; fair: 5; poor: 2 (FW)	Femoral shaft delayed union: 5; femoral shaft nonunion: 2; femoral neck varus malunion: 2
7.	Mahapatra et al. [18]	2017	18 (Group 1: 8; Group 2: 10)	Group 1: 23 (18–35) M; Group 2: 28 (20–32) M	Neck	Group 1: Type 1: 2; Type 2: 3; Type 3: 1; Type 4: 2; Group 2: (Type 1: 5; Type 2: 3, Type 3: 1, Segmental: 1	Group 1: CCS/DHS with distal femoral nail/biological plate fixation; Group 2: cephalomedullary nail (recon nail/long PFN)	Group 1: 15 (14–18) W; Group 2: NA	Group 1: 20 (14–28) W; Group 2: 23.4 (18–34) W	Group 1: Good: 6; fair: 2; Group 2: good: 7; fair: 1; poor: 2 (FW)	Group 1: femoral shaft delayed union: 2; Group 2: femoral head AVN: 1; femoral shaft delayed union: 2
8.	Lawson et al. [16]	2017	10	43.5 (6–108) M	Neck (4)/IT (6)	Type A: 6 (A2: 4, A3: 2); Type B: 2 (B1: 1, B2: 1); Type C: 2 (C1: 1, C2: 1)	Multiple	5.14 (3–12) M	5 (3–8) M	Good: 3; fair: 4; poor: 3 (FW)	Femoral neck nonunion: 1
9.	Gadegone et al. [13]	2013	36	NA	Neck (18)/IT (12)/subtrochanteric (4)	Proximal third: 4; middle third: 19; lower third: 13; Type 1: 14; Type 2: 10; Type 3: 6; Type 4: 4; segmental: 2	Long PFN	4.8 (4–8) M	6.2 (6–9) M	Good: 23; fair: 11; poor: 2 (FW)	Femoral shaft nonunion: 2; Femoral neck nonunion: 1; femoral head AVN: 1; shortening of 2 cm: 4
10.	Bali et al. [6]	2013	16	NA	Neck (13)/IT (3)	NA	Long PFN/recon nail	NA	NA	15 cases achieved satisfactory reduction	Proximal (hip) fracture nonunion: 1
11.	Ostrum et al. [7]	2013	92	23.92 (16–72) M	Neck (68 total; 2 subcapital, 13 transcervical, 53 basicervical)/IT (23)/ subtrochanteric-1	Comminuted (32C): 28; butterfly fragment (32B): 27; transverse (32A): 36; distal third (33A): 1	Hip screw (DHS/CCS) and retrograde reamed intramedullary nails	NA	NA	NA	Femoral neck nonunion: 2; femoral shaft nonunion: 4; femoral shaft delayed union: 3; knee pain: 10; hip pain: 2; chondromalacia patella: 3; both hip and knee pain: 1; B/L deep vein thrombosis: 1; knee flexion contracture of 5: 2; pulmonary embolism and septicemia: 1; abdominal compartment syndrome and acute renal failure: 1
12.	Kesemenli et al. [17]	2012	41 (Group 1: 24; Group 2: 17)	24 M	Neck (27)/IT (14)	AO Type A: 8 (A1: 4, A2: 4); Type B: 25 (B1: 13, B2: 7, B3: 5); Type C: 8 (C1: 5, C3: 3)	Group 1: CCS/DHS with plate (24); Group 2: cephalomedullary nails (17)	16 (14–23) W	Group 1: 31 (16–46) W; Group 2: 21 (15–33) W	Group 1: Good: 76%; fair and poor: 24%; Group 2: good: 83%; fair and poor: 17% (FW)	Group 1: femoral shaft nonunion: 7; femoral shaft implant failure: 3; femoral shaft delayed union: 4; superficial infections: 4; Group 2: femoral neck varus malunion: 2; delayed union: 1; wound infection: 1; shortening (<2.5 cm): 3
13.	Wang et al. [14]	2012	23 (Group 1: 13; Group 2: 10)	Group 1: 17.8 M, Group 2: 16.8 M	IT (23)	NA	Group 1: DHS and compression plate (13); Group 2: long PFN (10)	Group 1: 17.4 ± 3.11 (12–20) W; Group 2: 16.6 ± 3.13 (12–20) W	Group 1: 22.2 ± 4.2 (20–36) W; Group 2: 21.5 ± 2.66 (20–32) W	Group 1: Good: 9; fair: 2; poor: 2; Group 2: good: 8; fair: 1; poor: 1 (FW)	Group 1: deep infection: 1; femoral shaft nonunion: 2; Group 2: superficial infection: 1; femoral shaft nonunion: 1
14.	Bedi et al. [22]	2009	37	34.4 (12–112) M	Neck (21 displaced, 16 nondisplaced)	Type 1: 7; Type 2: 10; Type 3: 11; Type 4: 9	Cephalomedullary nail with or without CCS; CCS/DHS with retrograde femoral nail	14 out of 16 with more than 12 months of follow-up had united	All 16 with more than 12 months of follow-up had united	NA	Femoral neck nonunion: 2; femoral shaft malunion: 2
15.	Oh et al. [27]	2006	17	Until radiographs showed solid continuous callus formation	Neck	Type 1: 1; Type 2: 11; Type 3: 3; Type 4: 2; AO Type A: 13; Type B: 2; Type C: 2	Retrograde nail/CCS	11 (8–12) W	27.3 (14–60) W	Good: 16; fair: 1 (FW)	Femoral shaft nonunion: 5; femoral neck nonunion and femoral head AVN: 1, shortening of 1 cm: 1

Before the advent of reconstruction nails and PFN, conventional nails (K nails or femoral interlocking nails) were used for femoral shaft fracture, and CCS or other devices were used for femoral neck fracture [1,2,4]. During the early 2000s, various studies described the use of a single implant in the form of a reconstruction nail or second-generation cephalomedullary nail (PFN/PFN-antirotation) to address both fractures [6,8,9,13,19,21]. However, almost all of these studies stressed that this procedure is technically demanding and has a steep learning curve. The advantages found were minimal incisions and blood loss as well as biological fixation of both fractures. During the same period, some authors decided to move towards separate implants for better fixation [25,26], while others performed studies comparing the single implant versus double implant modalities of fixation [14,17,18,27,28]. These studies found no statistically significant difference with respect to union, functional outcome, or complications. However, it was determined that double implants require more extensive surgical exposure, prolonged operative time, increased cost, and the possibility of a stress riser in the area of the femur not spanned by any of the implants. Technically speaking, case series that used “miss the nail” screws along with conventional nails should also be considered double implants [4,9]. A newly introduced rendezvous technique, described by Harewood et al. [29], used an overlapping area of the nail and DHS plate, with one of the screws passing through both the plate hole and the nail. This technique prevents a stress riser in the area not otherwise spanned by either of the implants [27,29].

The opinion is divided among authors regarding the sequence of fixation. Most authors feel that femoral neck fixation takes precedence to avoid further injury to the femoral head blood supply [3,4]. However, others opine for fixation of the femoral shaft first to have better control during indirect reduction of the neck [20,29]. The hip fracture component of these injuries is mildly displaced or undisplaced [11,17]. This is due to the fact that in most of these fractures, the impact of trauma is borne by the femoral shaft and only that which is left by the femoral neck or intertrochanteric region [4,17]. If the femoral neck or intertrochanteric fracture is undisplaced, it can be provisionally fixed by a screw or by making passes with two thick K wires to prevent any displacement during surgery, followed by routine nailing [28]. If the femoral neck fracture is displaced, it is better to attempt closed reduction by applying a Schanz pin to the trochanteric fragment to achieve reduction. If that fails, the surgeon can either proceed with femoral nailing first and then attempt to reduce the femoral neck by traction on the fracture table, or the surgeon can attempt the open reduction of the femoral neck [22]. Although perfect reduction of the femoral neck is desirable and should be aspired for, two out of five femoral neck fractures in our series went on to unite despite nonanatomical reduction. This leads us to question whether open reduction of femoral neck fractures to achieve strict anatomical reduction, which substantially increases operative duration, is necessary.

One of the major complications noted in such fractures is nonunion of the femoral shaft. In our series, we had a high rate of nonunion (four out of 10 patients) with the delayed union in another two patients. Among these cases of nonunion, three were infra-isthmic and one was comminuted (at the isthmus level). This led to a gross mismatch between the size of the medullary cavity and the nail and hence inadequate fixation of the fracture. Seong et al. [21] determined that PFN was basically designed for intertrochanteric and subtrochanteric fractures and that distal locking bolts do not allow sufficient fixation in infra-isthmic-type fractures or even in isthmic fractures that are comminuted. They suggested the use of poller or blocking screws for satisfactory fracture reduction and biomechanical stability. They used this technique in 12 of their 27 cases, two of which resulted in nonunion, which limits the generalizability of their recommendation.

Wei and Lin [25] found that 78.6% (11 out of 16) of the patients with infra-isthmic fractures went into nonunion. The reason they cited was a “pendulum phenomenon” due to the mismatch between the larger medullary cavity of infra-isthmic fractures and the diameter of the nail. Antegrade nails cannot provide sufficient stability in these fractures and may result in nonunion. Retrograde femoral nails reduce this by allowing fixation in the metaphyseal part of the distal fragment and the proximal end well above the isthmic region [25]. However, it is important to note that a smaller diameter nail may lead to nonunion of the shaft, as pointed out by Oh et al. [27] and Ostrum et al. [7]. As these injuries are usually due to high-velocity trauma, femoral shaft fractures are comminuted, and an intramedullary implant can work at best as a load-bearing device but not as a load-sharing one in these fractures. Further, the “pendulum phenomenon” in infra-isthmic fractures puts stress on the distal screws, which may then fail. In our series, we had three such cases.

Nonunion of femoral neck fractures and AVN of the femoral head following these fractures, when associated with femoral shaft fractures, is quite low (2%) compared to isolated femoral neck fractures (10%) [7]. In our series, despite the nonanatomical fixation of these neck fractures in a few cases, the union eventually progressed. With intertrochanteric fractures, the union is usually not an issue, but varus positioning of the implant may lead to malunion in the varus. In one of the first meta-analyses published, Alho et al. [1] found that out of 659 cases, the rate of AVN was quite low (3%) compared to isolated femoral neck fractures (10%). In a series of 23 patients, Jain et al. [8] found three cases of delayed union and one each of non-union, AVN, and varus malunion. The explanation for why femoral neck fractures unite more readily when associated with femoral shaft fractures may be due to the fracture often being undisplaced or minimally displaced [4,11,17]. There may, however, be some biological reason behind this phenomenon, such as increased angiogenesis similar to what is done surgically by corticotomy during distraction osteogenesis. This may also explain the low incidence of AVN of the femoral head found in such cases. In our view, CT angiography-based studies should be conducted in the future to determine the amount of blood flow to the hip region following such concurrent fractures.

One aspect of such fractures that has often been neglected is their association with intra-articular ligamentous/meniscal knee injuries that may present later as knee stiffness or knee laxity. As dashboard injuries to the knee are one of the common modes of injury, we feel that this has been underreported in the literature. Due to disuse atrophy of the quadriceps and hamstrings in the period following the fracture, this may become more severe. Several authors have pointed out that poor functional outcomes in their series were mainly due to associated ipsilateral knee injuries [3,7,8,14]. Jain et al. [8] found major injuries to the knee in more than one-quarter of their cases. In their series of 95 cases, Ostrum et al. [7] found long-term knee pain to be present in 10 of their patients, while Casey and Chapman [3] found it in nine of their 62 patients. Kang et al. [26] determined that complications related to the knee are one of the major factors determining the outcome of dual-implant constructs in which retrograde femoral nails have been used. We recommend the use of titanium implants as these allow for an MRI at a later date, and thus it can be determined if surgery is needed to address these injuries.

The poorer outcomes found in our study compared to the previously published literature gave us an opportunity to go back and introspect on our findings. One of the major takeaways of our study is the increased chance of femoral shaft fractures going into nonunion when the location of the fracture is infra-isthmic. Femoral neck fractures uniting despite nonanatomic reduction makes us question whether performing open reduction is necessary. Due to the relative rarity of this disease, this study was limited by small sample size, retrospective design, and relatively short follow-up. Another major limitation was the fact that many of our patients had multiple injuries in other parts of the body, which had its bearing on the functional outcome and could have acted as a confounding factor. As these fractures are relatively uncommon, we propose a multicenter randomized controlled trial comparing different modalities of fixation with long-term follow-up to determine the natural history after fixation. The association of these fractures with intra-articular injuries to the knee should also be examined in future studies.

## Conclusions

Based on our study, we conclude that, although technically demanding, second-generation cephalomedullary nail is an acceptable and minimally invasive alternative for the management of concomitant ipsilateral fractures of the hip and femoral shaft, but only in those cases where the femoral shaft fracture is at the supra-isthmic (subtrochanteric) or isthmic level. It is not a good modality for infra-isthmic fractures, for which insertion of a retrograde femoral nail or distal femoral plate in a minimally invasive manner along with a separate implant for addressing the hip fracture (either CCS or if DHS, preferably in a rendezvous manner) are better options.
